# High‐Volume Processed, ITO‐Free Superstrates and Substrates for Roll‐to‐Roll Development of Organic Electronics

**DOI:** 10.1002/advs.201400002

**Published:** 2014-11-22

**Authors:** Markus Hösel, Dechan Angmo, Roar R. Søndergaard, Gisele A. dos Reis Benatto, Jon E. Carlé, Mikkel Jørgensen, Frederik C. Krebs

**Affiliations:** ^1^ Department of Energy Conversion and StorageTechnical University of DenmarkFrederiksborgvej 399DK‐4000RoskildeDenmark

**Keywords:** flexible materials, ITO‐free materials, printed electronics, superstrates, substrates, organic solar cells

## Abstract

The fabrication of substrates and superstrates prepared by scalable roll‐to‐roll methods is reviewed. The substrates and superstrates that act as the flexible carrier for the processing of functional organic electronic devices are an essential component, and proposals are made about how the general availability of various forms of these materials is needed to accelerate the development of the field of organic electronics. The initial development of the replacement of indium‐tin‐oxide (ITO) for the flexible carrier materials is described and a description of how roll‐to‐roll processing development led to simplification from an initially complex make‐up to higher performing materials through a more simple process is also presented. This process intensification through process simplification is viewed as a central strategy for upscaling, increasing throughput, performance, and cost reduction.

## 1 Introduction

The majority of current optoelectronic devices such as organic and polymer solar cells (OPVs) and organic light emitting diodes (OLEDs) are fabricated on glass carriers or small plastic sheets using a variety of fabrication processes for each of the functional layers including the electrodes, either semi‐transparent or opaque. The most used transparent conductive electrode and carrier material is the combination of indium tin oxide (ITO) and glass with a share of over 95% throughout the scientific community.^1^ Spin coating and subtractive patterning are commonly used for laboratory demonstrations and deliver impressive results for all the device‐specific parameters but most of the processes lack the scalability for an industrially relevant fabrication procedure. On the other hand, it is often claimed that the future of (organic) optoelectronic devices will be made on plastic and in large‐scale entirely through low‐cost roll‐to‐roll (R2R) processes using multidimensional solution‐based printing and coating processes. Only a limited number of reports exist where such an upscaled manufacturing procedure is presented for some of the functional layers.^2^, ^3^, ^4^, ^5^, ^6^ OPV devices in which all layers including front and back electrodes are fully R2R solution processed are still the minority.^7^, ^8^, ^9^, ^10^, ^11^ It should be mentioned that a lot of reports on organic (opto‐) electronic devices have been published that employ and describe fabrication methods other than spin coating such as gravure, inkjet, spray coating, and doctor blading, but the proof of the ultimate upscaling potential is very limited.^12^, ^13^, ^14^ So far, all reports on large‐scale processed devices are also far removed from the record‐breaking efficiencies, where the device sizes are most often significantly below 1 cm^2^ with power outputs too low to be usable.^1^ The most plausible reason is the lack of R2R equipment and the availability of active materials in high quantity, which is understandable, but also lack of suitable carrier materials with conductive electrodes (either transparent or opaque) in sufficient quantity, quality and layout to enable process development and fabrication of a reasonable amount of devices in large scale and area.

The development of R2R or sheet‐based large‐scale processes^15^ for any given organic electronics product will by nature require availability of patterned and conductive carrier material in significant amounts. Ideally rolls of several hundreds of meters to kilometers will be needed initially and in the case where one wishes to explore subsequent processes with web speeds of 10 m min^−1^ or higher many kilometers will be needed. In addition to a high cost being a limiting factor the dependence upon a supplier of the base material is a clear limitation. From this point of view processes and techniques for in‐house manufacture or outsourcing to local manufacturers of basic electrode structures are needed to ensure rapid development of processes and processing methods for printed electronics.

**Figure 1:**
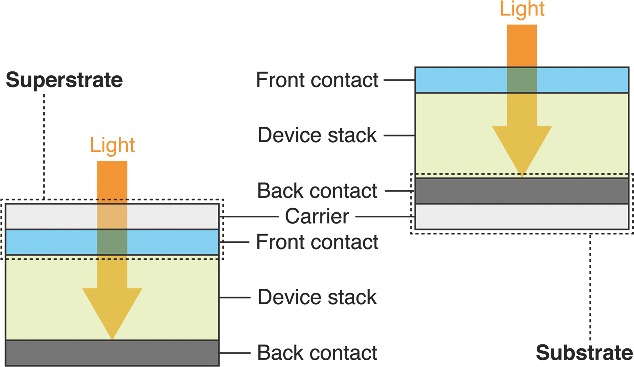
Simplified device stack of an OPV device to show the distinction between superstrate and substrate. Superstrates include a transparent conductive front contact, whereby substrates are the carriers with the back contact, either opaque or transparent.

To date, almost all of the available publications on organic optoelectronics devices use the term “substrate” for the carrier on which the electrode and device is built. Although this is technically not wrong, the functional layers are deposited on the carrier below (Latin sub = behind, under), it is often not the correct terminology in the final device as it is operated. In this case, substrates are behind the functional layer stack where the light does not necessarily need to pass through the substrate. It can be opaque and its main function is electrical conductivity. The majority of semitransparent conductive electrode carrier structures in optoelectronic devices such as OPVs or OLEDs act as superstrate (Latin super = above, on), which are located between the sun (eye) and the functional layer stack. Therefore the superstrate is the carrier combined with the transparent conductive electrode layer where the light is supposed to pass the functional film (i.e., in case of a solar cell) or to reach the eye of the user if emitted from the functional film (i.e., in the case of a light emitting device). A schematic of the distinction between sub‐ and superstrates for optoelectronic devices (for an organic solar cell) is illustrated in **Figure** **1**.

Here, we describe how kilometers of superstrates and substrates with semitransparent or opaque electrode structures can be prepared at high speed and we demonstrate how they can be used for manufacture of polymer solar cell devices and modules on a large scale. After a brief review of electrode structures in the literature, we describe the equipment needed, the ink quantities needed, and the time it actually takes to develop a new electrode structure suitable for large‐scale processing. Once the initial challenge for finding a process and an ink system for a given machine has been surmounted, we describe how simple it is to alter patterns to suit further development needs. We further assess each of the presented superstrate and substrate strategies with respect to their technical parameters and simplicity in fabrication. The manufacturing of sample devices on each of the electrode structures concludes this report.

## 2 Electrode Materials – the Current Status

Conductive carrier structures for optoelectronic devices are the fundamental element of any organic optoelectronic device. The most common material for the subsequent electrode deposition is rigid glass but materials such as polyethylene terephthalate (PET) or polyethylene naphthalate (PEN) in the form of thin flexible foils are the only that can be easily used in R2R machinery. ITO is the most used transparent conductive electrode for all of the organic optoelectronic devices and is used in 95% of all fabricated devices; it can be seen as the standard.^1^ The OLED and touchscreen market also demands a majority of ITO. The scarcity and localized mining of indium have led to fluctuations and a general increase in cost over the recent years and have opened the quest for alternative electrodes.^16^ Furthermore, the high embodied energy^17^ due to vacuum sputtering processes and the poor mechanical properties and brittleness^18^, ^19^, ^20^ has driven researchers to find new materials and processes with comparable or superior properties. In the case of a superstrate structure, the electrode should have a low sheet resistance combined with a high transparency to enable efficient current flow and large device areas. In case of flexible OPV devices with ITO‐PET superstrates the typical sheet resistance is in the range of 50 to 60 Ω sq^‐1^, which limits the cell sizes.^21^ ITO glass allows lower sheet resistances and is commonly used in laboratory‐scale test devices with limited upscaling potential.

A huge variety of alternative electrode structures for OPV superstrates have emerged, based on conductive polymers such as poly(3,4‐ethylene dioxythiophene):polystyrene (PEDOT:PSS), hybrid structures with metal grid and conductive polymers, metal nanowires (NW), ultrathin metals, carbon nanotubes, and graphene. An overview of optoelectronic devices with these superstrate electrodes is listed in **Table** **1**; the table only shows a selection of publications. The entire spectrum of transparent electrode superstrate structures is covered elsewhere in more detailed review.^22^, ^23^, ^24^ In case the device structure does not require a transparent conductive carrier the light has to pass the last deposited layer as previously shown in Figure 1. Hereby, the conductive substrate structure can be made from metal such as silver, or multilayer metal stacks from aluminum, titanium, and chromium on PET, glass, or paper. Steel has also been used as a conductive back contact layer that acts simultaneously as a carrier material in the form of steel foil. An overview with a selection of publications on organic optoelectronic devices with conductive opaque substrates is listed in **Table** **2**.

**Table 1:** Overview of several organic optoelectronic devices with transparent conductive superstrate carrier structures

Superstrate										
Carrier	Material	Device	*R*_s_ [Ω sq^‐1^]	*T* [%]	OPV eff. [%]	Methods, electrode	A active [cm^2^]	R2R?	Notes	Ref.
PET	AgNP	EL	4	95		IJ	0.2		coffee rings	25
PET	AgNP grid	EL	9	>75		Evap. litho				26
PET	MWCNT	EL	16 300	66.3		IJ, RC				27
Glass	Au grid	OLED	15	63		SC, Litho, EV	0.08		Au hex grid	28
Glass	AgNW, PEDOT:PSS	OLED	5.8	84		Spray, SC			laser patterning	29
PET	AgNP grid, PEDOT:PSS	OPV i	<12 (Ag)		1.7	IJ, SP	15.4	X	R2R flash	30
Glass	Graphene	OPV i	30	>85	>3.5					31
Glass	AgNW	OPV i	13–18	>90	2.3	SC	0.24			32
Polymer	AgNW	OPV i	16	82.3	3.07	SC, peel off	0.38		embedded	33
PET	Ag	OPV i	5	30	1.6	SD or SC	1	X	0.25% for full R2R module	34
PET	AgNP grid, PEDOT:PSS	OPV i	10.4	68	1.6	FL, SP	66	X	module	35
PET	AgNP grid, PEDOT:PSS	OPV i	<20	>60	2	FL, SP	147000	X	module	10
PET	AgNP grid, PEDOT:PSS	OPV i	10	82	1.92	R2R imprint, SP	6	X		36
PET	PEDOT:PSS	OPV i	220	80	3	SC	0.03			37
Glass	Ag mesh	OPV i	10	86	2.14	EV	0.09		Crack template	38
PET	Graphene, PEDOT:PSS	OPV n+i, OLED	<80	>80	>4.6	CVD, SC	0.126 (4)		etching and transfer	39
Plastic	Ag grid, PEDOT:PSS	OPV n	0.5 (Ag)	n/a	1	Soft litho, SC	0.08			40
Glass	Ag grid, PEDOT:PSS	OPV n	8.5 (Ag grid)	> 87	2.8	IJ, DB	0.09			41
PET	PEDOT:PSS	OPV n	230	75	2.2	SC	0.06			42
PEN	AgNP grid, PEDOT:PSS	OPV n	1 (Ag grid)		1.93	SP, SC	4		embedded	43
Glass	AgNP, PEDOT:PSS	OPV n	<<20		1.4	IJ	4			44
Glass	Mo, Al, Mo	OPV n	<<27		1.47	EV, IJ	4			44
PET	PEDOT:PSS	OPV n		90	2.8	SC	0.04			45
PET	PEDOT:PSS	OPV n	100		4.2	SC	0.1		PET 1.4 μm stretchable	46
PEN	AgNP grid, PEDOT:PSS	OPV n	4.8 (Ag)		1.54	IJ	4			47
PEN	AgNP grid, PEDOT:PSS	OPV n	500 (PEDOT:PSS)		1.38	IJ	4.92		Flash	48
Glass	PEDOT:PSS, GMS	OPV n	98	80	7.06	SC	0.1			49
Glass	PEDOT:PSS, ITO	OPV n	36	84	3.21	Spray, Sputter				50
Glass	AgNP grid, PEDOT:PSS	OPV n			1.94	IJ	0.09			51
PET, PEN	AgNP grid, PEDOT:PSS	OPV n	0.21 (Ag)	77.5	2.15	SP, SC	4	(X)	embedded	52
Glass	AgNP grid, PEDOT:PSS	OPV n		>77	2.54	IJ, DB	0.25		embedded	53
PET	AgNP grid, graphene	OPV n	11.5	74.5	2.9	IJ, CVD	0.046			54
Glass	Au grid, PEDOT:PTS	OPV n	<17 (Au)	>70	>3	EV, litho, SC	0.06			55
Glass	Graphene	OPV n	100 k–500 k	85–95	0.4	SC	0.008			56
PET	Graphene	OPV n	230	72	1.18	CVD	0.0075			57
PET	Cu mesh, PEDOT:PSS	OPV n	22	>70	2.1	NIL, EV	0.0078		R2R demo	58
P(VDF‐TrFE)	Graphene	OPV n	70	87	2.07	CVD, SC, etch, transfer				59
Glass	CuNiNW	OPV n	36	80	4.9	RC, Ni plating				60
Glass	Graphene	OPV t	521	70	8.02	SC, Litho	0.04		mesh	61
PET, PUA	AgNW, GO	PLED	14	88		RC, soaking	800		stretchable	62
PET	AgNP grid	EC	<5	82		Evap. litho			EC with WO_3_	63
PET	AgNP grid	EC				FL, SC	4	(X)		64

i = inverted, n = normal, t = tandem, GO = graphene oxide, NP = nanoparticle, NW = nanowire, MWCNT = multiwall cabon nanotube, EL = electroluminescent device, EC = electrochromic device, IJ = inkjet, RC = rod coating, EV = evaporation, SC = spin coating, GP = gravure printing, SD = slot‐die coating, SP = screen printing, CVD = chemical vapor deposition, NIL = nano imprint lithography, DB = doctor blading, FL = flexo printing, P(VDF‐TrFE) = poly(vinylidene fluoride‐co‐trifluoroethylene), PUA = polyurethane acrylate.

**Table 2:** Overview over several organic optoelectronic devices with opaque conductive substrate carrier structures

**Substrate**									
**Carrier**	**Material**	**Device**	***R*_s_ [Ω sq^‐1^]**	**OPV eff. [%]**	**Methods, electrode**	***A* active [cm^2^]**	**R2R?**	**Notes**	**Ref.**
Glass	Al, Ag	OLED			EV	0.06			65
Glass	Al, Ti	OPV i		3.4	EV	0.08			66
PET	Al, Ti	OPV i		1.8	EV	4		metal wrap through	66
Steel	Ag	OPV i		1.73	EV	0.01			67
Glass	Ag	OPV i		2.5	EV	0.02			68
PET	Cr, Al, Cr	OPV i		2.2	Sputter	13.2			69
Paper	Zn, ZnO	OPV i		1.3	GP, transfer	0.09	X		8
Paper	Zn, ZnO	OPV i		4.1	GP, transfer	0.1	(X)	Optim. polymer + structure	70
PET	Cr, Al, Cr	OPV i		2.9	Sputter	1.1		shadow mask	3
PEN	AgNP	OPV i	0.01	0.3	SD	120	X	module	71
PET	Ag	OPV i	<1	2.6	SD	1	X		72
Glass	Al	OPV n		3.17	EV	25		monolithic	73
PET	Al	OPV n		2.8	EV	25		monolithic	74
Steel	Steel	OPV n	0.5	1.3		50		1 cm^2^ illum.	75
Paper	Au	PD			EV				76

The majority of conductive electrodes requires sputtering, evaporation, or spin coating and requires patterning processes for proper device manufacturing. Masked evaporation or subtractive post‐processes for full layer coatings lead to material waste and prevents efficient upscaling. Although some of the alternative electrodes show impressive parameters, the fabrication is very challenging or devices are processed only on very small areas in the range of several square millimeters. Most of the electrodes are made in limited quantities only for the scientific experiment and the upscaling potential is often questionable or pending several developments in other areas. ITO is not highlighted in the overview because it is the standard superstrate and is commercially available. It can be purchased on glass, PET, etc. and structuring can be carried out in the lab through etching or laser ablation processes or made on request by the supplier, e.g., using R2R etching, stripping, and cleaning. In all cases a subtractive process is used and material is wasted.

The most efficient way to fabricate a conductive carrier material is by using only additive steps through defined printing or coating processes. In this case the functional material is only deposited where it is necessary and material waste can be fully avoided or minimized to a very low fraction with respect to fabrication volume. The highest output is expected through fast R2R processes. Possible large‐scale methods for conductive structures include flexo‐printing,^35^, ^77^, ^78^, ^79^ gravure printing and coating,^80^, ^81^, ^82^ rotary screen printing,^35^, ^83^ inkjet printing,^47^, ^84^, ^85^ or a variety of coating processes including slot‐die coating, all of which can be performed in a full R2R process^34^, ^71^, ^86^, ^87^, ^88^ Embedding conductive structures inside the carrier material is an interesting method to smooth the layer but needs more complex equipment in a full R2R process.^52^, ^89^

## 3 Superstrates and Substrates for Everyone

This very brief review shows that there is much ongoing research in the field of conducting electrodes but most of the scientists still use ITO because no cheap alternatives are available in sufficent quantity that can be patterned entirely through additive processes. To show relevant results on large‐scale processed devices the fundamental conductive substrate or superstrate is the first requirement, in addition to the availability of a highly efficient active layer material in large quantity. We believe that potential replacements for ITO and the methods of making them have to be made available to everyone in high volume; otherwise ITO will be replaced very slowly or perhaps never. Because the efficiencies of large‐area and large‐scale processed OPV devices are still low it requires very large areas to generate useful power output.^10^ Even costly metals such as silver should be avoided or minimized, although it was shown that silver is highly recyclable.^90^ Supporting metal grids can be justified for the optimization of current collection in larger single cells and for interconnection to modules,^10^, ^91^ but it might be unnecessary with a proper device design depending on the application.^92^, ^93^

**Figure 2:**
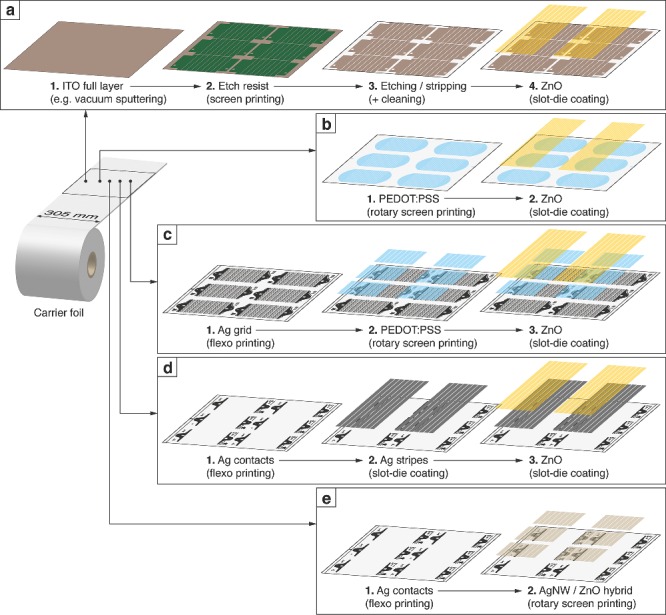
Fabrication workflow of patterned electron accepting superstrates with a) ITO/ZnO, b) PEDOT:PSS/ZnO, and c) silver grid/PEDOT:PSS/ZnO. d) An opaque full silver/ZnO substrate with additional printed silver contact electrodes. ZnO acts as electron transporting layer in all electrode configurations. The ultimate process simplification is reached with a hybrid AgNW/ZnO superstrate electrode that can be printed in a single printing step (e). Only outside contact are printed separately to improve device contacting.

**Figure 3:**
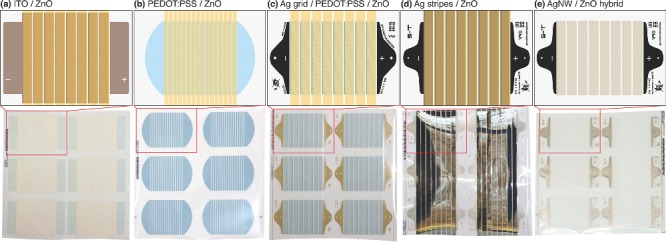
Postcard‐sized module layout of the patterned superstrates with a) ITO, b) just PEDOT:PSS, and c) silver grid/PEDOT:PSS as conductive layer. An opaque substrate module layout with additional contact electrodes is shown in (d). The electron transporting ZnO layer is slot‐die coated in panels (a–d). The hybrid AgNW/ZnO superstrate electrode (e) is fully printed without using slot‐die processes. The top row shows a more detailed graphical illustration of the corresponding photographs of full 12” × 12” motifs in the bottom row.

The fabrication steps and workflow of the proposed flexible superstrates and substrates with electron transport characteristics that we developed and process in our labs under ambient conditions are shown in **Figure** **2**. Corresponding illustrations of a single module pattern with the size of a typical postcard are shown in **Figure** **3**. The current designs are based on web widths of 305 mm and have six modules deposited per motif length of 12”. The carrier material is a roll of pure PET or barrier foil when wishing to avoid further encapsulation of the corresponding device side. Fabrication of patterned ITO with fixed conductivity and transmittance, shown just for comparison, requires the most process steps and involves subtractive etching processes with high material loss. The entire process is typically outsourced to a specialized supplier who demands a desired pattern in digital form to fabricate screen printing masks for applying a positive mask before etching. The fabrication speed is rather slow and requires specialized machinery for handling the chemicals. Basic electrical and optical parameter change aside from the layout requires the etching of completely new rolls of ITO foil.

A superstrate with just two process steps is illustrated in Figure 2b, where highly conductive PEDOT:PSS is rotary screen printed and hole conducting ZnO is slot‐die coated with a small lateral offset to enable contacting. Silver can be avoided due to 16 cells with a smaller width of just 4 mm, hereby the high fill factor (FF) is retained, instead of 8 cells with 10 mm width. The geometric fill factor also decreases. The active areas in a final module are in the range of 57 cm^2^ for the 8‐cell device or ca. 30 cm^2^ for the 16‐cell device. A supporting grid structure is not required for such small cell widths if sufficient highly conductive PEDOT:PSS is used. Outer electrodes for the final module connection are directly printed in the first step and coating of ZnO only needs lateral registration.^93^

The improved version for larger cell sizes employs an additional flexo‐printed grid structure of silver nanoparticle (AgNP) ink prior to PEDOT:PSS and is shown in Figure 2c. Grid structures can range from hexagonal to diamond grid, or even parallel grid fingers, depending on the application and electrical layout. Our current version is based on 5° slanted grid fingers in the direction of the current flow and a grid pitch of 1.5 mm. Flexo‐printing allows very fine structures below 100 μm. Outer electrodes are flexo‐printed together with the grid electrode, while PEDOT:PSS is just printed in rectangular patterns over the individual cells. The PEDOT:PSS printing processes requires registration both in lateral and web directions. ZnO is slot‐die coated in continuous stripes as usual with a slight lateral offset.^11^

OPV devices in a substrate structure do not require optical transmittance of the back contact. Full additive processing of slot‐die coated reflecting silver stripes made from nanoparticle‐free silver ink is shown in Figure 2d and Figure 3d. Studies also showed the possibility to R2R gravure print the full silver electrode stripes using commercial silver ink but the surface quality was poor compared to the slot‐die coated electrode, mainly due to limitations in coating speed (2 m min^−1^). External AgNP contacts and registration marks are flexo‐printed prior to slot‐die coating and allow for easy contacting in a final OPV module. Slot‐die coated ZnO finalizes the electron conducting layer stack. Interestingly, the substrate can be easily transformed to a transparent superstrate stack by diluting the silver ink. Hereby the silver layer becomes semitransparent with some loss of conductivity.^34^ The philosophy of pre‐printed silver contacts and slot‐die coated stripes of conducting ink is very practical for the fabrication of semi‐patterned electrode structures for optoelectronic devices. Some inks, such as AgNW, CuNW, or special self‐assembling ink mixtures, are still difficult to print directly into the necessary patterns (beyond stripes) and are often tailored for pure coating processes.

Nevertheless, we developed a process that allows printing AgNW and ZnO in a single run by using a hybrid ink mixture as illustrated Figure 2e and Figure 3e. This ultimate workflow simplification enables the fabrication of electron transporting superstrate electrodes in just one printing step while the outer contact silver electrodes are flexo‐printed beforehand to allow proper device connection. They could of course be eliminated, thus enabling realization of a Flextrode in a single step (as opposed to three steps that are normally required for standard Flextrode).^35^ Our ink formulations also allow separate printing of each component. Avoiding slot‐die coating and using only printing processes results in the best material utilization for large‐scale fabrication because the material is only deposited where it is necessary. The slot‐die coating of ZnO in all other electrode workflow processes described before results in areas with unused material coverage. The hybrid process truly follows the “print only where needed” principle and allows free‐form electrode layouts that are not limited to the well‐known stripes from slot‐die coating.

## 4 Experimental Workflow and Methods

Full layer ITO superstrate foil is produced through sputtering and used for further patterning that involves masking with etch resist, etching with aqueous copper chloride (or ferric chloride), stripping with sodium hydroxide, and washing with demineralized water. The exact process parameters such as fabrication speed are either confidential or not fully available. In the case of large scale manufacture of patterned ITO the overall process speed is significantly less than 1 m min^−1^. Prior studies also revealed that ITO has a huge share of embodied energy with close to 87% in a final OPV device, which can be reduced significantly by using alternatives to ITO.^94^

The four replacements presented here are fabricated via all‐additive processes with parameters listed in **Table** **3**. The fundamental carrier foils employed here were either PET (Melinex ST506, 125 μm) or Amcor barrier foil (50–70 μm). The three fabrication methods that are combined in different ways are flexo‐printing (FL), rotary screen printing (RSP), and slot‐die coating (SD), whereby the electrode fabrication workflow is illustrated in Figure 2. In brief, flexo‐printing relies on a soft printing form where the raised parts define the image. Ink is transferred from a so‐called anilox cylinder with a defined ink volume to the soft printing form and from there onto the foil using nip‐induced surface interactions. Rotary screen printing employs a cylindrical mesh, in which the open parts define the printing pattern. An internal squeegee forces the ink or paste through the rotating mesh onto the foil. The wet layer thickness and printing definition are mainly defined by the mesh parameters. Slot‐die coating employs a coating head with an internal ink distribution chamber, feed slot, and mask (shim) for coating stripes. The wet layer thickness of such a pre‐metered process is based on ink flow rate, coating speed and coating width. All of the described printing and coating processes can go beyond 100 m min^−1^, whereas the speeds in these studies are limited to around 10–20 m min^−1^ due to limited drying length (hot air and infrared (IR)) of the employed R2R equipment. Further details and photographs of the R2R machinery and individual process steps can be found elsewhere.^12^, ^35^, ^95^

**Table 3:** Process parameter for the R2R fabrication of ITO‐free superstrates and substrates

Material	Method	Speed [m min^‐1^]	Thickness [nm]	Drying
**PEDOT:PSS/ZnO (superstrate**, Figure 2b**)**				
PEDOT:PSS (Clevios PH1000 : IPA 10:3)	RSP	>10	≈400 (dry)	140 °C hot air + IR
ZnO in acetone	SD	10	≈100 (dry)	70/140 °C
**AgNP grid/PEDOT:PSS/ZnO (superstrate,** Figure 2c**)**				
AgNP (Nanopchem PFI‐722)	FL	>20	≈200 (dry)	140 °C hot air + IR
PEDOT:PSS (Clevios PH1000 : IPA 10:3)	RSP	>10	≈200 (dry)	140 °C hot air + IR
ZnO in acetone	SD	10	≈100 (dry)	70/140 °C
**AgNP contact/Ag full/ZnO (substrate,** Figure 2d**)**				
AgNP (Nanopchem PFI‐722)	FL	>20	≈200 (dry)	140 °C hot air + IR
Ag (Kunshan Hisense SC‐100 : IPA 1:1)	SD	2	≈100 (dry)	140 °C hot air + IR
ZnO in acetone	SD	10	≈100 (dry)	70/140 °C
**AgNP contact/AgNW/ZnO hybrid (superstrate,** Figure 2e**)**				
AgNP (Nanopchem PFI‐722)	FL	>20	≈200 (dry)	140 °C hot air + IR
AgNW/ZnO hybrid	RSP	15	≈100 (dry)	140 °C hot air + IR

OPV test cells and modules were fabricated using our standard procedures on a mini rollcoater^96^, ^97^, ^98^ using slot‐die coating of active layer and PEDOT:PSS electrode together with a flexo‐printed Ag grid electrode, or in a full R2R process with slot‐die coated active layer, rotary screen printed PEDOT:PSS, and rotary screen printed silver or carbon paste electrode.^11^, ^93^

Transmittance and reflectance measurements were performed on a Shimadzu UV‐3600 UV‐VIS spectrometer. Reflectance and the transmittance for the AgNW/ZnO hybrid electrode were measured using an integrated sphere to collect all diffused and scattered light. Air was used as reference in all cases and transmittance values include the specific carrier substrate. The sheet resistances have been measured using a Jandel RM3 4‐point station. Bending tests were carried out on a Mecmesin Multitest 2.5‐i test bench and custom made data acquisition software to measure the resistance of electrode after each bend. The bending diameter for strain and compression tests was 10 mm. Solar cells were measured with a Keithley 2400 sourcemeter under a KHS 1200 solar simulator with an AM1.5G 1000 W m^−2^ intensity.

## 5 Results and Discussion

### 5.1 Optical Transmittance and Reflectance

**Figure 4:**
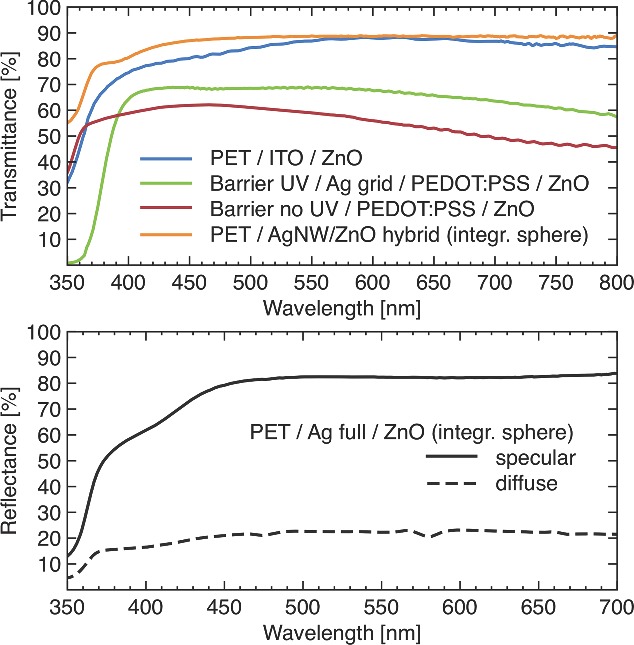
Transmittance (top) and reflectance (bottom) of five different electron conductive electrodes on a variety of carriers.

All superstrates have in common that their transmittance in the visible range is high while the substrate described here has high specular reflectivity. The comparison of UV‐vis spectrometer measurements for all electrodes is shown in **Figure** **4**. The transmittance was measured for electrodes fabricated on a variety of carrier materials that have variable optical qualities. Normalization to the pure electrode was neglected because a real device cannot be made without a carrier that of course contributes to the final device performance. It also shows the variability of the electrode fabrication on different carriers. Furthermore, barrier foil was used with and without UV blocker, and therefore the region of interest for all transmittance values is from 400 nm and higher. The highest transmittance is presented by the AgNW/ZnO hybrid electrode with more than 88% transmission at 550 nm, which is better than ITO/ZnO along the entire visible spectrum. It presents a very optically neutral behavior with iridescence due to the AgNW. The plasmonic resonance of the AgNW also improves the transmittance and was shown to be higher than the geometric aperture.^99^ This electrode was measured using an integrating sphere to collect all scattered light. The other electrodes showed no substantial difference in the transmittance for measurements with or without integrating sphere.

The blue colored PEDOT:PSS based electrodes exhibit the typical drop in transmittance in the higher wavelengths and NIR range that makes them potentially less efficient for OPV devices with low bandgap polymer in the active layer. The electrode with supporting Ag grids has a transmittance close to 70% while the Ag grid‐free electrode has a 10% lower transmittance. The main reason can be found in the thicker PEDOT:PSS layer to achieve a sheet resistance of 44 Ω sq^‐1^ comparable to ITO. PEDOT:PSS electrodes with Ag grid support can tolerate less conductive and thinner films because the highly conductive Ag grid has the highest contribution to the conductivity in the electrode stack.

The opaque substrate studied here is a highly reflective electrode with a specular and diffuse reflectance >80% and >20% at 550 nm, respectively. This mirror‐like behavior acts as backside reflector in an OPV and can largely contribute to an improved efficiency of the respective device.^72^

### 5.2 Flexibility

**Figure 5:**
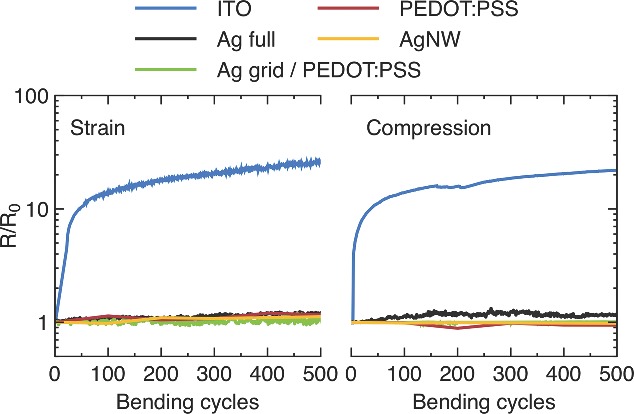
Bending test results for the electrodes shown for strain and compression over 500 bending cycles. The measured resistance *R* is normalized to the initial reference value *R*_0_.

Flexible devices require highly flexible electrodes to tolerate mechanical stress not only during handling but also during the device fabrication using R2R machinery. ITO is known for its brittleness, which is also reflected here in the fast increase in sheet resistance after a couple of bending cycles as shown in **Figure** **5**. The other electrodes are superior under bending stress and show no major performance loss even after 500 cycles.

### 5.3 Process Workflow and Electrode Parameters

The best electrode with highest optical parameters and lowest sheet resistance is worthless if it is not processable on a large‐scale for the desired application, such for OPV or OLED devices. The material usage and energy input during the fabrication has a high impact on the economy of the final device and should be considered from the beginning. Many incredible procedures for the fabrication of transparent electrodes can be found in the literature but most of them are only of academic value and are impossible (at least financially) to produce on a large scale. Despite the fact that patterning is often ignored, it would require subtractive processes that results in unnecessary material loss.

Our additive fabrication workflows shown in Figure 2 for ITO‐free electrodes just require ordinary printing and coating equipment, which enables even local print shops to produce such printed electronics products without the need for making investments in highly specialized equipment. Research facilities investigating the processing of optoelectronic devices are also able to produce their own electrodes without dependence on an external supplier of etched ITO. Adjustments to the pattern or layout of the electrode can be made relatively fast to suit their own needs with a printing form supplier on hand. The density of such external services is much higher than finding an ITO etching service nearby. The key to the working electrode is the functional ink, of which the majority are already commercially available and optimized for the specific printing processes.

A comparison of the main characteristics of the five electron collecting electrode stacks in superstrate and substrate architecture is listed in **Table** **4**. It is shown that all additive workflows require fewer process steps than the fabrication of patterned ITO with just two steps to the electron transport layer ZnO. The AgNW/ZnO hybrid electrode can be printed in just one step if the outer printed electrode is neglected. It also waives the use of slot‐die coating and makes true free form devices possible.

**Table 4:** Comparison of the main characteristics of the five electron collecting electrode stacks in superstrate and substrate architecture

	ITO/ZnO	Ag grid/PEDOT:PSS/ZnO	Ag full/ZnO	PEDOT:PSS/ZnO	AgNW/ZnO hybrid
Stack acronym		Flextrode	T2	SF	FLT
Type	Superstrate	Superstrate	Substrate	Superstrate	Superstrate
Additive	–	+	+	+	+
Printing required	+	+	+	+	+
Coating required	+	+	+	+	–
Vacuum required	+	–	–	–	–
Design freedom	– (stripe‐like)	– (stripe‐like)	– (stripe‐like)	– (stripe‐like)	+ (any shape)
# of steps to ETL (incl. outer contacts)	4	3	3	2	2
# of steps to ETL (excl. outer contacts)	4	3	2	2	1
Potential bifaciality	+	+	–	+	+
Transmittance including carrier (550 nm)	>86%	>68%	NA	>58%	>88%
NIR transmittance	+	–	NA	–	+
Iridescence	–	–	–	–	+
Sheet resistance	≈50 Ω/sq	<20 Ω/sq	≈575 mΩ/sq	≈44 Ω/sq	<20 Ω/sq
Flexibility	–	+	+	+	+

Although all processes of the alternative electrodes are additive, they do involve material waste from the printing and coating processes. This can be substantially minimized when the continuous fabrication output is larger. The material waste due to unused ink and cleaning is the same if 10 m are printed or when or 10 km are printed, considering that all processes are optimized and controlled. The relative wastage is obviously much lower for large outputs than for small ones. Subtractive processes will have the same relative material waste independently of the output size and eventually require expensive recycling processes for all the material lost during patterning.

### 5.4 Device Examples

**Figure 6:**
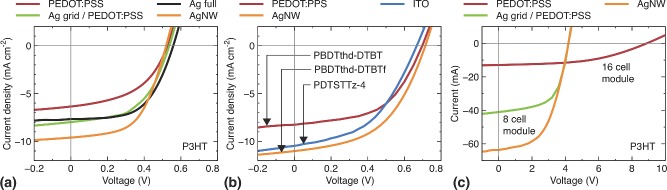
*J*–*V* curves of selected single cell devices with P3HT as a) donor polymer and b) low bandgap polymer fabricated on the mini‐rollcoater. c) *I*–*V* curves of modules fabricated entirely through R2R processes.

A selection of relevant *J*–*V* and *I*–*V* curves and parameters of sample OPV devices can be found in **Figure** **6** and **Table** **5**, respectively. The active layer polymers used were either standard P3HT or high performance low bandgap polymers in conjunction with PCBM as acceptor. The variety of devices, either R2R processed or laboratory‐scale‐sized on the mini‐rollcoater, show their broad applicability in research and development and in industrialized methods.

**Table 5:** Solar cell characteristics of selected devices fabricated on superstrates and substrates presented here

**Single cells, on mini‐rollcoater**						
Conductive electrode	Polymer	*V*_OC_ [V]	*J*_SC_ [mA cm^−2^]	FF [%]	PCE [%]	Area [cm^2^]
PEDOT:PSS	P3HT	0.52	‐6.35	48.4	1.61	0.2
PEDOT:PSS	PBDTthd‐DTBT^100^	0.69	‐7.64	52.9	2.82	0.4
Ag grid/PEDOT:PSS	P3HT	0.53	‐7.98	52.3	2.24	0.7
Ag full	P3HT	0.55	‐7.67	57.3	2.45	1.4
AgNW	P3HT	0.52	‐9.6	55.1	2.75	0.71
AgNW	PBDTthd‐DTBTf 100	0.71	‐11.01	49.7	3.9	0.7
ITO	PDTSTTz‐4^101^	0.67	‐10.46	47.1	3.29	0.8
**Modules, R2R processed**						
Conductive electrode			*I*_SC_ [mA]			
PEDOT:PSS	P3HT	8.88	‐12.96	47.7	1.83	30 (16 cells)
Ag grid/PEDOT:PSS	P3HT	4.2	‐41	60	1.82	57 (8 cells)
AgNW	P3HT	4.19	‐63.65	52.7	2.46	57 (8 cells)

The efficiency for ITO superstrate devices with P3HT:PCBM as active layer and full R2R processing following the ProcessOne routine are in the range of 1.7–2.3% depending on the device area and module configuration.^7^ The device design was slightly different to the post‐card sized module layout presented here but layer stack and fabrication conditions are similar.

First studies on R2R processed silver and ITO free devices based on rotary screen printed PEDOT:PSS layers were already performed earlier with the main purpose of life cycle assessment studies.^92^ Efficiencies of 1.6% could be achieved on slightly smaller credit‐card sized devices and an active area of 15.4 cm^2^.^83^ Single test devices manufactured on a mini‐rollcoater and the current superstrate resulted in an efficiency of 1.61% for P3HT:PCBM and >2.8% for a low bandgap polymer without any further device optimizations. Fully R2R processed carbon‐based modules completely without silver demonstrated efficiencies up to 1.8% based on an active area of 30 cm^2^. The statistical studies and further details on a dataset of 500 modules can be found elsewhere.^93^

The superstrate with Ag grid/PEDOT:PSS/ZnO, also known as Flextrode, achieved roughly 1.8% with P3HT:PCBM as active layer fabricated in a full R2R process. The modules known as freeOPV have (at the time of writing this account) been handed out to >9000 interested people.^11^ Essentially all our recent publications are based on this superstrate type and a variety of different OPV devices have been fabricated including tandem devices,^102^, ^103^ modules with efficiencies up to 3.2%, and single cells of 3.8%,^98^, ^100^, ^101^ The same superstrate stack but different module layout was used for the fabrication of very large and scalable modules with active areas beyond 14 m^2^ and power outputs >250 W_peak_.^10^, ^104^ Life cycle assessment calculations showed very promising energy paypack times <180 days for entire systems including mounting scaffolds.

The Flextrode superstrate itself is also available free of charge to all academics^35^ and was already used by others to fabricate dye‐sensitized solar cells (DSSC) with efficiencies beyond 6%.^105^ In this case the electrode comprised only AgNP grid/PEDOT:PSS. Surface improvements due to ozone and plasma treatment have been found to be important for work function recovery of the ZnO layer after long storage time.^106^

The opaque substrate based on reflective silver has been successfully used to fabricate single P3HT:PCBM cells on the rollcoater with an efficiency of 2.45% (area 1.4 cm^2^) and high fill factor >57%, where the silver layer simultaneously acts as back reflector and conductor. The best P3HT:PCBM cell achieved a PCE of 2.6% with slightly lower fill factor. The reflecting silver substrate is also suitable for fully solution‐processed tandem solar cell devices with efficiencies beyond 2.35% on an area of 0.8 cm^2^. More detailed studies on the silver layer and device fabrication have been published elsewhere.^72^

The hybrid AgNW/ZnO superstrate electrode has been used for the fabrication of functional OPV devices with efficiencies close to 4% on active areas of around 1 cm^2^. Cells and modules were fabricated using small‐scale roll coating equipment and large‐scale R2R equipment, respectively. The device examples clearly show that all electrodes can be used either for full R2R production of OPV modules or for device preparation of small test cells using laboratory equipment. Hereby, pieces of electrodes are cut from the mother roll and used for further fabrication of devices to emulate large‐scale processes. The achieved efficiencies of polymer solar cells fabricated under industrially relevant processes on the alternative electrodes without the use of vacuum are compatible or superior to ITO‐based devices. The availability of kilometers of transparent electrodes on cheap carrier material is a necessity to prove R2R compatibility of new materials and device configurations.

## 6 Conclusion

We have introduced the distinction between superstrates and substrates based on the entire device stack of the optoelectronic device. If the carrier and electrode is viewed alone, without any application in mind, the substrate nomenclature is fully justified. We showed that the described superstrates and substrates are comparable or superior to ITO in electrode‐specific parameters but also with respect to large‐scale manufactured devices under ambient conditions. The results show that use of ITO is not necessary anymore, and all‐additive fabrication routes are now state‐of‐the‐art. The fabrication employs standard printing and coating processes that in principle can be fabricated by the local printing and coating industry or in‐house if the required R2R equipment is available. Everybody is invited to test the available superstrates and substrates for their application and improve subsequent processes, and we believe that general (and free) availability is key to both progress and finally replacing ITO.
